# Impact of Thermal Treatment and Aging on Lignin Properties in Spruce Wood: Pathways to Value-Added Applications

**DOI:** 10.3390/polym17020238

**Published:** 2025-01-18

**Authors:** František Kačík, Eva Výbohová, Tereza Jurczyková, Adriana Eštoková, Elena Kmeťová, Danica Kačíková

**Affiliations:** 1 Department of Chemistry and Chemical Technology, Faculty of Wood Sciences and Technology, Technical University in Zvolen, 96001 Zvolen, Slovakia; vybohova@tuzvo.sk; 2 Department of Wood Processing and Biomaterials, Faculty of Forestry and Wood Sciences, Czech University of Life Sciences Prague, Kamýcká 129, 16000 Prague, Czech Republic; jurczykova@fld.czu.cz; 3 Institute for Sustainable and Circular Construction, Faculty of Civil Engineering, Technical University of Košice, Vysokoškolská 4, 04200 Košice, Slovakia; adriana.estokova@tuke.sk; 4 Department of Fire Protection, Faculty of Wood Sciences and Technology, Technical University in Zvolen, 96001 Zvolen, Slovakia; xkmetovae@is.tuzvo.sk (E.K.); kacikova@is.tuzvo.sk (D.K.)

**Keywords:** spruce wood, thermal treatment, lignin, nitrobenzene oxidation, thermogravimetry, size exclusion chromatography, infrared spectroscopy, vanillin, life cycle

## Abstract

Thermal modification is an environmentally friendly process that does not utilize chemical agents to enhance the stability and durability of wood. The use of thermally modified wood results in a significantly extended lifespan compared with untreated wood, with minimal maintenance requirements, thereby reducing the carbon footprint. This study examines the impact of varying modification temperatures (160, 180, and 210 °C) on the lignin of spruce wood using the ThermoWood process and following the accelerated aging of thermally modified wood. Wet chemistry methods, including nitrobenzene oxidation (NBO), size exclusion chromatography (SEC), thermogravimetry (TG), differential thermogravimetry (DTG), and Fourier transform infrared spectroscopy (FTIR), were employed to investigate the alterations in lignin. At lower modification temperatures, the predominant reaction is the degradation of lignin, which results in a reduction in the molecular weight and an enhanced yield of NBO (vanillin and vanillic acid) products. At elevated temperatures, condensation and repolymerization reactions become the dominant processes, increasing these traits. The lignin content of aged wood is higher than that of thermally modified wood, which has a lower molecular weight and a lower decomposition temperature. The results demonstrate that lignin isolated from thermally modified wood at the end of its life cycle is a promising feedstock for carbon-based materials and the production of a variety of aromatic monomers, including phenols, aromatic aldehydes and acids, and benzene derivatives.

## 1. Introduction

Global climate change requires reducing the production of greenhouse gases, which can be significantly helped by the widely spread and abundantly used raw material—wood. Wood is created in photosynthesis and subsequent reactions from carbon dioxide, which can be re-released into the atmosphere when wood is burned or can be bound and stored in wood and its products.

Among the many wood-processing and modification technologies, heat treatment is very important. It is environmentally friendly, using only elevated temperatures and water, without adding other chemicals. This process improves wood properties, producing a material, which, at the end of its life cycle, does not present an environmental hazard that is higher than untreated wood. At the present stage, it is necessary to consider the environmental performance of the thermally modified products and end-of-life scenarios to create strategies after the end of the life of products, including recycling and disposal [1,2,3]. Thermal modification improves the dimensional stability, microbial durability, and deteriorates some mechanical properties, which is closely related to chemical changes in the main wood components. Thermal degradation is accompanied by the emission of volatile products (water, carbon dioxide, methanol, formaldehyde, carboxylic acids, etc.) created by the degradation of extractives, polysaccharides, and lignin. Acetyl groups are cleaved from hemicelluloses, which in the form of acetic acid catalyze the depolymerization of hemicelluloses and the amorphous part of cellulose [4,5,6].

The necessity for sustainable development and the reduction in the reliance on fossil-based industries for chemicals, fuels, fuel additives, and energy have underscored the significance of harnessing the individual components of lignocellulose, each with its unique potential for diverse product streams [7]. Lignin is an irregular polyphenolic biopolymer constructed of phenylpropanoid monomers with various degrees of methoxylation that are biosynthesized into a complex and highly heterogeneous aromatic macromolecule [8].

Despite the initial challenges associated with the robust and irregular structure of lignin, the valorization of this intriguing aromatic biopolymer has come a long way. Recently, many creative strategies have emerged that deliver defined products via catalytic or biocatalytic depolymerization in good yields [9]. The choice of a suitable method of the further processing of lignin depends on its structure, which changes during wood modifications.

Spruce milled wood lignin (MWL) is significantly branched and cross-linked (~36% of lignin units are partaking in these linkages). About half of the branching and crosslinking linkages involve aromatic rings, predominantly 5–5′ etherified units; meanwhile, many linkages are in the side chains. The branches involve different aliphatic ether (alkyl-*O*-alkyl) types at the α- and γ-positions of the side chain, with intact β-*O*-4 linkages [10]. It is the β-*O*-4 linkages that play a major role in obtaining vanillin from lignin. Vanillin is originally obtained from vanilla beans and is widely used in the food and pharmaceutical industries. However, the mentioned source cannot cover the need for vanillin, and currently, most of the vanillin is produced from the petrochemical raw material, guaiacol. However, lignin is a more desirable raw material for vanillin production compared with guaiacol due to its unique aromatic structure, high reserve, and low price. In addition, vanillin made from lignin is more environmentally friendly and economical than guaiacol. The yields of vanillin from lignin are affected by interunit linkages and the results show that the more β-*O*-4 linkages in lignin, the higher the yield of corresponding aldehydes obtained [11].

**Figure 1:**
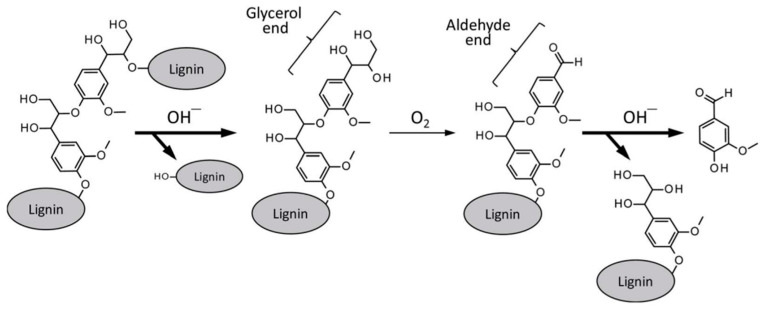
Vanillin formation from a β-*O*-4 middle unit of a lignin polymer [12].

The formation of vanillin and vanillic acid from wood involves complex chemical transformations primarily driven by the degradation of lignin, a major component of the plant cell wall. Lignin degradation can occur through various processes, including oxidative depolymerization, acid-catalyzed reactions, and thermal treatments, which yield aromatic compounds such as vanillin and vanillic acid. Figure 1 shows one of the schemes of vanillin formation.

Vanillin (4-hydroxy-3-methoxybenzaldehyde) is a primary product of lignin degradation. Its formation can occur through the oxidative cleavage of lignin’s aromatic structures, particularly those containing guaiacyl units. Studies have shown that vanillin can be produced in significant yields (5–15 wt%) during the oxidative degradation of lignin using metal oxides and hydrogen peroxide as catalysts [13]. The oxidative processes often involve the cleavage of β-*O*-4 linkages, which are prevalent in lignin, leading to the release of smaller phenolic compounds, including vanillin [14].

Vanillic acid (4-hydroxy-3-methoxybenzoic acid) is another important product that can be formed from vanillin through further oxidation. Research indicates that vanillin can undergo oxidation reactions, particularly in the presence of oxygen or oxidizing agents, to yield vanillic acid. However, it is important to note that while vanillin oxidation can yield vanillic acid, most vanillic acid obtained during lignin oxidation may be produced via alternative pathways [15]. Acetic acid, released from hemicellulose during hydrothermal treatments, can also catalyze these reactions, facilitating the conversion of lignin-derived compounds into vanillic acid [16]. In addition to oxidative pathways, acid-catalyzed reactions play a significant role in forming these compounds. For instance, the use of *p*-toluenesulfonic acid at moderate temperatures has been shown to promote the valorization of wood while minimizing lignin condensation, thus enhancing the yield of valuable products like vanillin and vanillic acid [17]. The controlled application of acids can help in breaking down lignin without leading to extensive repolymerization, which is a common challenge in lignin processing [18].

Thermal treatments, such as pyrolysis, also contribute to the formation of vanillin and vanillic acid. During pyrolysis, the degradation of lignin occurs at elevated temperatures, leading to the release of various volatile compounds, including aromatic aldehydes and acids [19]. The thermal degradation of lignin can be optimized to favor the production of these compounds by controlling the temperature and reaction environment, thereby the minimizing condensation reactions that would otherwise lead to higher-molecular-weight products [20].

The modification of lignin during thermal treatment is a complex process influenced by factors such as temperature, heating rate, and atmosphere. Understanding the structural transformations that lignin undergoes under these conditions is essential for harnessing its potential. Thermal treatment can lead to the depolymerization of lignin, breaking its complex structure into simpler compounds. This can enhance the reactivity of lignin and open possibilities for its utilization in value-added products [21,22]. Norway spruce lignin has relatively high β-*O*-4 linkages (44.7 per 100 C_9_ units) [23], creating prerequisites for high vanillin yields.

**Figure 2:**
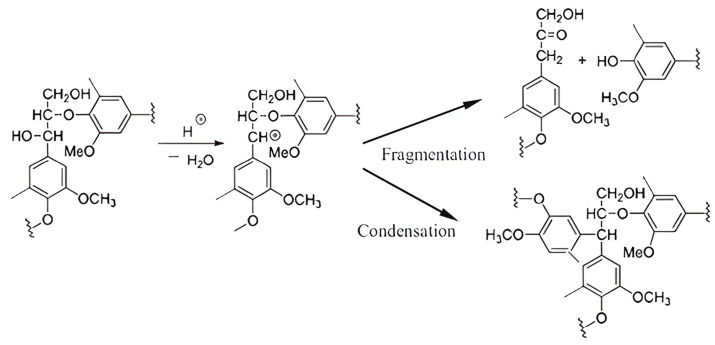
Reaction scheme of the lignin β-*O*-4 structure during acidic pretreatment, denoting the competition between depolymerization and condensation [29].

Changes in lignin during heat treatment has been investigated in quite some detail. With increased heat, the lignin polymer undergoes several cleavage reactions, self-condensation, polycondensation, and cross-linking to form lignin–lignin and lignin–carbohydrate complexes (Figure 2). These lead to a relative increase in the lignin content in wood [24]. The condensation of lignin derivatives is a significant concern during degradation processes. High temperatures and prolonged reaction times can lead to the repolymerization of lignin fragments, resulting in the formation of complex, condensed structures that are difficult to process [25,26]. This phenomenon underscores the importance of optimizing reaction conditions to minimize condensation while maximizing the yield of the desired products. For example, studies have indicated that lower reaction temperatures can enhance the yield of phenolic compounds by reducing the likelihood of further degradation and condensation [25]. Changes in lignin depend not only on the temperature and treatment time, but also on the type of wood, and even under the same modification conditions, different reactions take place in the lignins of various wood species [27,28].

The influence of accelerated aging on the changes in the main components of wood has also been investigated in relative detail. Still, information on the accelerated and natural aging of thermally modified wood is rare [30,31]. The aging reactions of wood lignin are critical to understanding its stability, reactivity, and potential applications in various industries. Lignin, a complex biopolymer, undergoes a series of chemical transformations over time, influenced by environmental factors such as temperature, moisture, and exposure to oxygen. These aging reactions can lead to changes in lignin’s structure and properties, impacting its functionality in applications such as biofuels, materials science, and as a natural antioxidant. One of the primary aging reactions of lignin is oxidative degradation, which can occur through various mechanisms, including forming free radicals. This process can lead to the cleavage of ether and carbon–carbon bonds within the lignin structure, generating smaller phenolic compounds [32]. Thermal aging is another significant factor affecting lignin stability. Studies have shown that heating lignin can induce degradation and condensation reactions. For instance, at elevated temperatures, the cleavage of methoxyl groups and the formation of cross-linking radicals can occur, leading to changes in the molecular weight and structural integrity [33,34]. The thermal treatment of lignin can also result in the formation of chromophore structures, which contribute to discoloration and affect the overall appearance of lignin-containing materials [35]. These thermal reactions are particularly important in processes such as pyrolysis, where lignin is converted into bio-oil and char. Furthermore, the aging of lignin can lead to the formation of complex structures, such as dibenzodioxins, which are formed under kraft pulping conditions. These structures can undergo further reactions, resulting in the generation of biphenyl compounds [36]. The presence of such compounds indicates that lignin aging is not merely a degradation process but also involves the formation of new, potentially valuable chemical entities. The antioxidant properties of lignin also play a crucial role in its aging reactions. Lignin’s phenolic structures enable it to act as an effective antioxidant, which can mitigate oxidative damage in various applications, including polymer composites and food products [37,38]. This antioxidant capacity can help to stabilize lignin-containing materials against oxidative aging, thereby enhancing their durability and performance.

This research focuses on isolated lignin transformation from thermally modified and accelerated aged wood, such as its degradation or condensation during heat treatment or its oxidative modifications during aging. Previous studies on this topic [16,21,35] lack a comprehensive analysis of the combined effects of thermal modification and aging on the molecular structure and reactivity of lignin. Furthermore, there is a limited understanding of the correlations between these structural changes and lignin’s potential in value-added applications, such as producing aromatic monomers. This study addresses these gaps by investigating the chemical, physical, and structural transformations of lignin in thermally modified spruce wood subjected to accelerated aging. Using advanced techniques, including nitrobenzene oxidation and size exclusion chromatography, we provide detailed insights on the molecular behavior of lignin. The results reveal critical pathways for optimizing lignin utilization at the end of its life cycle and offer novel strategies for producing high-value compounds and carbon-based materials. This work not only advances the scientific understanding of lignin dynamics but also supports the broader goal of sustainable material development.

## 2. Materials and Methods

### 2.1. Samples Preparation

The experiments were performed on spruce (*Picea abies*, Karst) from the central region of Czechia. The material consisted of five logs, each 2 m long. The logs were cut at a tree height of 1.3 m above the ground. The log diameters were from 0.4 to 0.5 m. The logs were processed into radial timber, 30 mm in thickness, which was gradually dried to a 16% moisture content. Then, from each log, four radial samples with the dimensions 200 mm × 100 mm × 20 mm were obtained. The specimens were sampled in such a way as not to contain juvenile wood. The samples were assorted in four sets and acclimated to a 12% moisture content. One sample set was subjected to thermic treatment at 160 °C, the second at 180 °C, and the third at 210 °C. The fourth set served as the control without treatment. After the thermal treatment, the test specimens were prepared from each sample set, as necessary.

### 2.2. Thermal Modification

The untreated group was denoted as REF. The remaining three groups were thermally modified following the ThermoWood process according to Sikora et al. [27].

1. Heating and drying. In this phase, the temperature in the oven increased to approximately 100 °C. Throughout this stage, the wood is dried to approximately zero moisture content.
2. Thermal modification. In the second phase, the temperature is increased to the desired level (160 °C, 180 °C, and 210 °C, respectively). After reaching the desired temperature, the oven is kept in a steady state and the actual treatment takes place for 3 h. During the thermal modification, the timber is protected using steam.
3. Cooling and climatization. In the third phase, the wood is gradually cooled to 80–90 °C, and the moisture content of the final product is 4–7%.

### 2.3. Accelerated Aging

The accelerated wood aging was simulated in a xenotest chamber Q-SUN Xe-3-HS (Q-Lab Europe, Ltd., Bolton, UK) the standard ASTM G155 [39]. The wood was aged using a “wet mode” to simulate the outdoor conditions where wood is exposed to UV radiation and rain. One accelerated aging cycle consisted of two steps, covering 120 min altogether; the total duration of the aging process was 600 h, equivalent to 300 cycles. Thermally treated and aged samples were denoted as 160-TW-XE, 180-TW-XE, and 210-TW-XE.

### 2.4. Chemical Analyses

Before the analysis, the whole untreated (REF), heat-treated (TW), and aged (TW-XE) samples were reduced manually to chips and further disintegrated and milled to a particle size of 200–300 μm using a POLYMIX PX-MFC 90D laboratory mill (Kinematica, Luzern, Switzerland) and dried (4 h at 103 ± 2 °C). They were then extracted using the Soxhlet apparatus (Sigma-Aldrich, Munich, Germany) using absolute ethanol–toluene solution (1.0/0.427 *v*/*v*) [40]. Klason lignin was determined using the NREL procedure [41]. Dioxane lignin was isolated using the previously published method [34]. Dioxane lignin samples were analyzed using attenuated total reflectance Fourier transform infrared spectroscopy (ATR-FTIR) and Size Exclusion Chromatography (SEC) methods.

### 2.5. Nitrobenzene Oxidation

Alkaline nitrobenzene oxidation (NBO) was carried out in a stainless-steel bomb using the modified method of Kačíková et al. [34]. NBO products were analyzed using high-performance liquid chromatography (HPLC) in an Agilent 1260 Infinity II Prime LC (Agilent, Santa Clara, CA, USA) apparatus equipped with a diode array detector at 210 nm, a Luna Omega column of 1.6 µm C18 (100 × 4.6 mm) (Phenomenex, Torrance, CA, USA), mobile phase water/methanol/propionic acid (88/4/8/0.1), a flow rate of 0.4 mL·min^−1^, and a temperature of 35 °C. The quantification was performed with the external calibration using standards (*p*-hydroxybenzaldehyde, *p*-hydroxybenzoic acid, vanillin, vanillic acid, syringaldehyde, and syringic acid) purchased from Sigma-Aldrich (St. Louis, MO, USA). NBO was performed in duplicate, and each sample was analyzed twice. Identification was made using a comparison of the retention times and UV spectra with the standards.

### 2.6. Size-Exclusion Chromatography

The molecular weight distribution (MWD) of dioxane lignin was measured according to the modified method of Zinovyev et al. [42] and Pittman et al. [43] using an Agilent 1200 HPLC chromatograph (Agilent Technologies, Santa Clara, CA, USA) with 0.5% LiBr in dimethylformaldehyde as mobile phase on three POLAR-M columns (7.5 mm × 300 mm, Agilent Technologies, Santa Clara, CA, USA). Dry samples were dissolved in the mobile phase at 2.0 mg·mL^−1^, and the flow rate was 1.0 mL·min^−1^ at 35 °C. The lignin samples were prepared in duplicate and chromatographed in two replicates.

### 2.7. Thermal Analysis

Thermal analyses were carried out using the simultaneous thermal analyzer NETZSCH STA 449 F3 (NETZSCH-Gerätebau GmbH, Selb, Germany). The experiments were performed in a nitrogen atmosphere at a 25–800 °C temperature range, with a heating rate of 10 °C min^−1^ using Al_2_O_3_ as a crucible material. The data were acquired and analyzed using the NETZSCH Proteus software (Version is 8.0.3).

### 2.8. ATR–FTIR Analysis

Dioxane lignin samples were analyzed using attenuated total reflectance Fourier transform infrared spectroscopy (ATR–FTIR). The spectra were acquired by accumulating 64 scans at a spectral resolution of 4 cm^−1^ in an absorbance mode from 4000 to 650 cm^−1^ and standardized using the baseline method. The obtained data were analyzed using the OMNIC 9.0 software. Measurements were performed four times per sample.

### 2.9. Statistical Evaluation

Statistical evaluation of the measured data was performed using the TIBCO Statistica software version 14 (TIBCO Software Inc., Palo Alto, CA, USA) for the indicated statistical methods: correlation analysis (Pearson’s correlation coefficient) to find out which parameters under investigation (VAN, VANac, *M*n, *M*w, *M*z, PDI) are related to each other; linear fit in 2D scatterplots to illustrate the dependence of the found correlations; and GLZ (ANOVA) analyses for testing the differences between individual groups of samples (thermally modified at different temperatures, generally thermally modified, and thermally modified and aged). From the outputs of the mentioned methods, only graphs are used for illustrative comparisons.

## 3. Results and Discussion

### 3.1. Extractives and Lignin Yields

Most of the original extractives are lost or degraded with the increasing process temperature and/or treatment time, but new extractable compounds are formed due to the thermal degradation of carbohydrates, mainly hemicelluloses, and, to a lesser extent, lignin [31,44,45].

**Figure 3:**
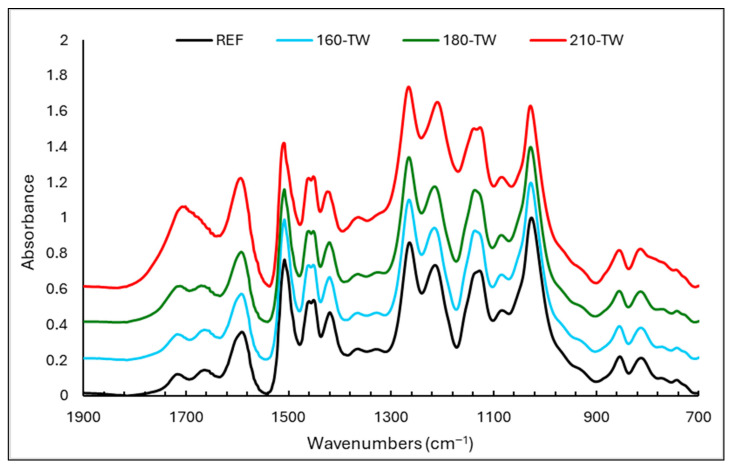
FTIR spectra of lignin isolated from untreated and thermally treated spruce wood.

**Figure 4:**
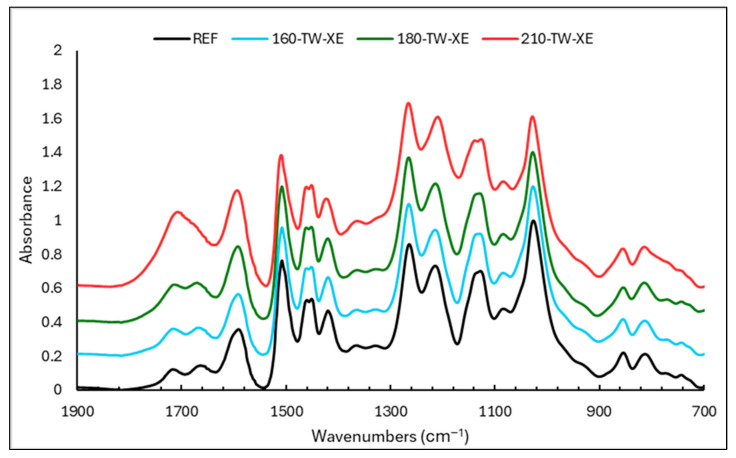
FTIR spectra of lignin isolated from untreated and thermally treated and aged spruce wood.

Extractive substances also significantly affect the color of the wood, which can be used as a quality indicator of Norway spruce and Scots pine subjected to the Thermo-D ThermoWood process [46,47]. In our experiments, the content of the extractives increases with the modification temperature, the smaller content is in the aged wood, probably due to the leaching of water-soluble substances by the action of water during the “wet mode” aging (Table 1). An increase in the content of lignin during thermal treatment was observed by several authors [27,48], which is due to the greater thermal stability of lignin to carbohydrates and the formation of pseudo-lignin, which contains polyfuran, aromatic, carbonyl, and aliphatic structures [49,50,51]. The lignin content in the reference sample is in good agreement with the results of other authors, e.g., 36.4% [52] and 28% [53]. In our work, a greater increase in lignin was observed during the aging process, probably due to the extraction of water-soluble substances during the wet-aging mode (Table 1). This observation is consistent with the increasing condensation of lignin analyzed by the size exclusion chromatography (SEC) method (Table 3; Figure 3 and Figure 4).

### 3.2. Nitrobenzene Oxidation Products

The main lignin recovery strategies focus on transforming lignin into valuable compounds that can be used in various industries. Oxidation with various reagents is often used for depolymerization; from an analytical point of view, oxidation with nitrobenzene in an alkaline environment is among the most commonly used. The main (frequently the only) product of the nitrobenzene oxidation (NBO) of conifer lignins is vanillin, usually used as a flavoring and fragrance ingredient in the food or cosmetic industries [54].

In our work, we found only the presence of guaiacyl structural units (vanillin and vanillic acid) (Table 2), which agrees with the findings of Wang et al. [11]; however, traces of *p*-hydroxybenzaldehyde and syringaldehyde were also detected in spruce lignin [7,55].

With NBO, softwood lignin gives rise to vanillin as the major product, usually in the range of 17–28% based on the Klason lignin content [52,56]; in pine lignin, 18.5% vanillin and vanillic acid has been determined by NBO [57]. Both degradation and condensation reactions occur in lignin during thermal treatment. The increase in the yield of NBO products at a temperature of 160 °C indicates the predominance of degradation reactions, as indicated by the decrease in molecular weight determined by the SEC method (Table 3; Figure 3 and Figure 4). At higher treatment temperatures, the yields decrease due to the predominance of condensation reactions, which agrees with the data of Kim et al. [21]. According to Wang et al. [11], less condensed lignins give higher yields of aldehydes in NBO. The type of bond is also important for the structural changes of lignin. More β-*O*-4 bonds in lignin give a higher yield of the corresponding aldehydes. It is believed that the yield of vanillin can be significantly improved by using lignins with a high content of β-*O*-4 bonds. At higher temperatures, these bonds are cleaved, lignin is demethoxylated, and its repolymerization occurs, leading to a decrease in the NBO yield [57,58]. Higher yields of NBO products after the accelerated aging of thermally modified wood may be due to lignin degradation and the cleavage of inter-unit bonds.

### 3.3. Molecular Weight and Molecular Weight Distribution of Lignins

The average molecular weight (MW) and molecular weight distribution (MWD) are important macromolecular properties of lignins because their reactivity and physicochemical properties are partly governed by their MWD [59]. Even though lignin begins to decompose due to heat already at low temperatures, compared with carbohydrates, it is the most thermally stable wood component, and its relative content increases after heat treatment [60,61]. Structural changes in lignin during modification include the cleavage of methoxyl groups and the depolymerization of the lignin macromolecule into lower molecular weight compounds and subsequent repolymerization. SEC analyses show (Table 3) a slight decrease in the molecular weight of lignin up to a temperature of 180 °C, followed by an increase. The drop in molecular weight is caused by the cleavage of various C−O bonds of the C3 side chain and especially the β-(*O*-4) ether bond [62]. At the same time, reactive intermediates such as carbonium ions, which can be formed during the cleavage of the benzylic C–O bond, participate in the recondensation reactions [63].

At lower temperatures and/or shorter treatment times, lignin degradation reactions prevail; higher temperatures and/or prolonged treatment cause repolymerization reactions to increase the molecular weight [64,65]. Our results show that depolymerization, side chain cleavage, and recondensation occur during heat modification. During the thermal modification of spruce wood, lignin depolymerization occurs at a temperature of 160 °C, and high-molecular lignin fractions are degraded. From a temperature of 180 °C, condensation reactions prevail, and lignin has more high-molecular fractions. This phenomenon is particularly noticeable at a temperature of 210 °C. Robles et al. [3] also published similar trends for pine wood lignin after thermal treatment at 212 °C and after service life. However, changes in the molecular weight of lignins during thermal treatment also depend on the wood species. For example, teak and merbau lignins depolymerize at a temperature of 160 °C, with condensation occurring at 180 °C and 210 °C, respectively. The trend is the opposite in the case of iroko and padauk lignins. Meranti lignin depolymerizes at all the temperatures applied [28,34].

**Table 3:** SEC results of spruce wood lignin (reference: REF, thermally treated: TW, and accelerated aged: XE samples).^a,b^

*T* (°C)	*M*_n_ (Da)	*M*_w_ (Da)	*M*_z_ (Da)	PDI
REF	2689 (29)	7397 (60)	18,164 (102)	2.75 (0.01)
160-TW	2983 (65)	6875 (82)	17,779 (156)	2.31 (0.02)
180-TW	2670 (33)	7165 (50)	15,758 (98)	2.68 (0.02)
210-TW	2744 (19)	7666 (66)	20,961 (164)	2.79 (0.04)
160-XE	2506 (31)	6590 (45)	14,101 (40)	2.63 (0.02)
180-XE	3116 (66)	7034 (88)	17,825 (102)	2.26 (0.02)
210-XE	2904 (46)	7638 (88)	21,015 (205)	2.63 (0.01)

^a^ Standard deviation values are in parentheses. ^b^ *M*_n_ = number average of molecular weight (MW). *M*_w_ = weight-average MW, *M*_z_ = **z** average MW, *M*_z + 1_ = z + 1 average MW, PDI (polydispersity index) = *M*_w_/*M*_n._

### 3.4. Thermal Analysis of Lignins

As confirmed in Table 4, the weight percentage of residual char decreases at a thermal treatment temperature of 160 °C and then increases. The temperatures of the main decomposition area (344–374 °C) have a similar trend, and at lower decomposition temperatures, a greater mass loss can be observed (Table 4). This suggests that, at lower temperatures, depolymerization reactions prevail and when the temperature increases, the lignin becomes more complex and establishes a condensed form with an increased C-C bond ratio [66]. These observations align with those reported by Kim et al. [21]. The course of degradation and repolymerization reactions is also supported by our nitrobenzene oxidation and size exclusion chromatography results and the published data [67,68,69]. In the case of aged wood samples, decomposition peaks also appear on the DTG curves at higher temperatures (359 °C for sample 180-TW-XE and 429 °C for sample 210-TW-XE), which indicates the possibility of condensation reactions of lignin due to UV radiation [70].

### 3.5. FTIR Analyses

From the point of view of vanillin yield, the key absorption bands belonging to β-*O*-4 bonds in lignin lie at the wavelengths of 1215 and 1137 cm^−1^ (Figure 3 and Figure 4). Therefore, their intensities in the lignin spectra will correspond with the determined vanillin yields (Table 2). However, several absorption bands overlap at the indicated wavelengths, which belong to different bonds or fragments of the lignin macromolecule. These are subject to changes during thermal modification and subsequent UV radiation, affecting the intensity of the given absorption bands in different directions and to different extents. For example, the band at 1215 cm^−1^ reaches a maximum in samples treated at 210 °C, whereas, on the contrary, the lowest yield of vanillin was determined. In addition to the vibrations of the C-O bond, the intensity of this band is determined to a greater extent by the stretching vibrations of C=O, the marked increase of which has been proven with an increasing treatment temperature. A better agreement can be observed between the yield of vanillin and the intensity of the absorption band at 1137 cm^−1^, although even at this wavelength, in addition to the vibration of aryl–ether bonds, aromatic C-H in-plane deformations typical of G units are also manifested.

The ether bond is readily cleaved, which leads to lignin depolymerization and the formation of monomers; in spruce wood, it is mainly guaiacyl structural units. This increases the phenolic hydroxyl groups’ content, which is reflected in the FTIR spectrum by an increase in the intensity of the absorption band at 3410 cm^−1^. Some authors have reported a relationship between hydroxyl groups and the molecular weight of lignins. A clear correlation was found between the lower *M*_n_ and the increased phenolic OH group content [71,72]. The low-*M*_w_ lignin fractions of spruce and eucalyptus lignins contained the largest number of phenolic hydroxyls and the lowest number of aliphatic hydroxyls [73,74].

Successive oxidation reactions of coniferyl alcohol form vanillin and vanillic acid, or lead to oxidation of the aromatic ring and form quinones [75,76]. Quinones are the main structures inducing color changes during the thermal treatment of wood. The increase in absorbance at 1717 cm^−1^ confirmed the formation of these products during both processes: thermal treatment and subsequent UV irradiation. The higher the treatment temperature, the higher the intensity of the carbonyl group band. Subsequent UV irradiation increased the height of the carbonyl band only slightly. In addition, it was observed that the higher the temperature of the previous thermal modification, the lower the intensity of the formation of new carbonyl groups during the subsequent UV irradiation.

### 3.6. Statistical Analysis

**Figure 5:**
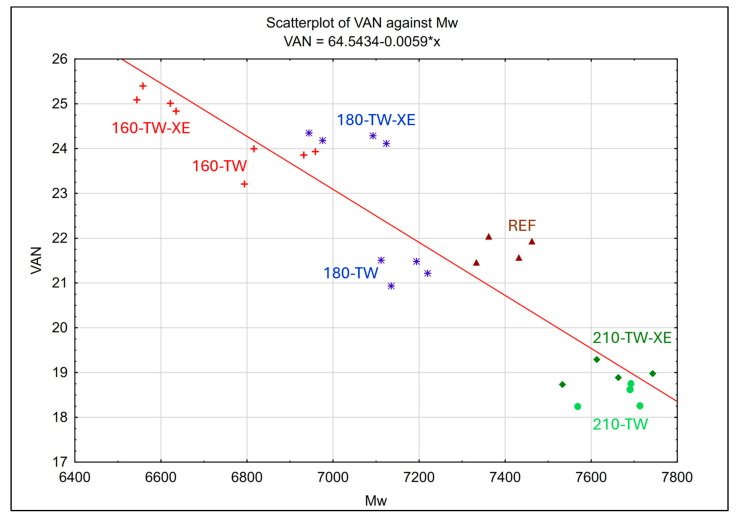
Linear dependence of the vanillin content (VAN) in wood lignin on its weight average molecular weight (*M*_w_).

According to the correlation coefficient −0.9255 (Table 5), a statistically significant relationship is observed between the content of determined vanillin (VAN) in lignin and the weight average molecular weight (*M*_w_) of lignin (Figure 5). *M*_w_ considers the molecular weight of a chain in determining its contribution to the molecular weight average. The bigger the chain, the more it contributes to *M*_w_. For the reference sample (REF), the average *M*_w_ ≈ 7397 Da and the VAN content is 21.75%. The closest to this is the 180-TW sample (*M*_w_ ≈ 7165 Da and VAN ≈ 21.29%), which has only slightly lower values of both the monitored parameters, i.e., there is a slight depolymerization, but with suppressed vanillin production. In the same sample subjected to the xenotest, i.e., 180-TW-XE, only a negligible difference in the value of *M*_w_ (a decrease of only 1.8%) is detected. Still, within the tested series before and after the xenotest, there is demonstrably the highest difference in the increase in vanillin content (by 13.8%) in this sample. Generally, the highest vanillin content (VAN ≈ 25.1%) was observed in the sample 160-TW-XE, i.e., an increase of 15.4% compared with REF. The sample of 160-TW-XE also has the lowest *M*_w_ ≈ 6590 Da, i.e., a decrease of 10.9% compared with REF. Before the xenotest, 160-TW had the second lowest *M*_w_ ≈ 6875 Da among all the tested samples and only a slightly lower vanillin content (VAN ≈ 23.75%) compared with the sample 180-TW-XE. The graph in Figure 5 shows that in the sample groups 160-TW, 160-TW-XE, and 180-TW-XE, there are distinct degradation reactions, during which vanillin is formed. On the other hand, the sample groups 210-TW and 210-TW-XE have a much lower content of vanillin (in the range of 12.8–15.1%) and higher *M*_w_ (by 3.3–3.6%) compared with REF, which confirms the presence of condensation and repolymerization reactions with the reduction in vanillin production compared with the REF sample.

**Figure 6:**
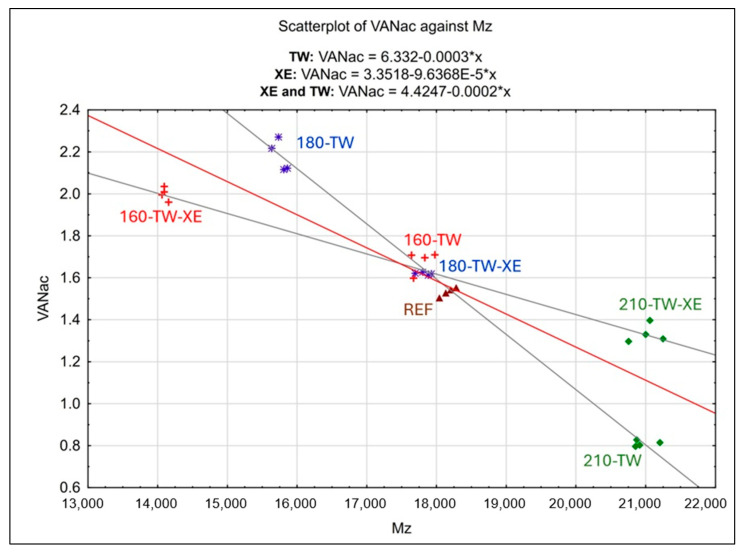
Linear dependence of the vanillin acid content (VANac) in wood lignin on its Z-average molecular weight (*M*_z_).

The graph in Figure 6 shows a second significant dependence, with a correlation coefficient of –0.8864 (Table 5) between vanillin acid (VANac) in the lignin sample and the Z-average molecular weights (*M*_z_) of wood lignin. Compared with *M*_w_, *M*_z_ is increasingly more sensitive to high-molecular-weight polymers. The reference sample (REF) provides average *M*_z_ ≈ 18,164 and VANac ≈ 1.53% values. In the same cluster, there are also samples of 160-TW and 180-TW-XE, which have only slightly lower *M*_z_ (≈ 17,779 Da and 17,825 Da, respectively) and marginally higher VANac (≈1.68% and 1.62%, respectively) values. This indicates a mild course of depolymerization reactions with an insignificant release of vanillin acid from the samples. These degradation reactions are observed to a greater extent in the case of the 180-TW and 160-TW-XE samples. The 180-TW sample has the highest vanillin acid content of all the tested samples (VANac ≈ 2.18), i.e., 42.5% more than REF, and its *M*_z_ ≈ 15,758 Da, i.e., 13.2% lower than REF. On the contrary, the 160-TW-XE sample has the lowest *M*_z_ ≈ 14,101 of all the tested samples, i.e., 22.4% lower than REF. In the samples, 210-TW and 210-TW-XE, the same trend concerning REF is observed as in the case of the previous dependence of VAN-*M*w: an increase in the molecular weight, in this case *M*_z_, and a decrease in the formation of degradation products, here, specifically VANac.

**Figure 7:**
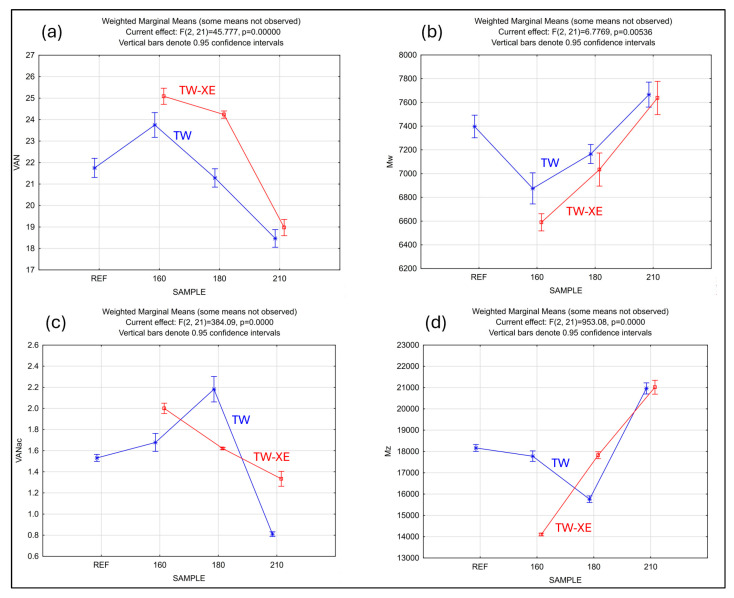
Results from GLZ (ANOVA models) for the different measures of dependence on sample and modification (models with interactions are used).

Both Figure 5 and Figure 6 show that the largest decrease in the molecular weights (*M*_w_ and *M*_z_) occurs in the 160-TW-XE sample, which also contains the highest proportion of vanillin. With the increasing temperature of thermal modification of the samples, the amount of vanillin decreases and the samples subjected to a xenotest always have a vanillin content that is 2.7–13.8% higher than their unaged state in the range of the thermal treatments examined (Figure 7a). With the increasing temperature, the thermal modification of sample *M*_w_ increases from 160 °C almost linearly after an initial decrease compared with the REF. In the case of the accelerated aged samples (TW-XE), *M*_w_ is always slightly lower (by 0.4–4.1%) compared with its unaged state (Figure 7b).

The highest proportion of vanillin acid is contained in the sample 180-TW. Viewed in terms of VAN-*M*_w_ dependence (Figure 5), and thus the average contributions to molecular weights, this 180-TW sample appears to be stable and resistant to degradation reactions. However, let us also consider the situation of VANac-*M*_z_ (Figure 6) influenced by high-molecular chains. There is a significant reduction in *M*_z_ due to their absence and evidence of degradation associated with depolymerizing lignin structures to form predominantly VANac. The nonlinear trend of the behavior of thermally treated (TW) samples versus aged (TW-XE) samples depending on the VANac modification temperature in Figure 7c, and the dependence of *M*_z_ on the sample modification temperature in Figure 7d, indicate a different degradation mechanism of thermally modified and thermally modified and aged samples at 180 °C: 180-TW samples probably have shorter chains in which there is a more significant release of vanillin acid. On the other hand, mild condensation reactions (which are demonstrable in the 210-TW and 210-TW-XE samples) associated with a decrease in VANac, but higher VAN production compared with the 180-TW sample, could be expected in the 180-XE samples. In both cases, condensation reactions of a similar magnitude related to an increase in the molecular weights (*M*_w_ and *M*_z_) and a decrease in the vanillin and vanillin acid content compared with REF are confirmed for samples 210-TW and 210-TW-XE (Figure 5 and Figure 6).

## 4. Conclusions

The focus of the study is to investigate the chemical, physical, and structural changes in the lignin of spruce wood subjected to thermal modification and accelerated aging, with the goal of understanding the effects of these processes on lignin properties. The study highlights the potential of aged thermally modified lignin as a feedstock for various value-added products, such as aromatic monomers and carbon-based materials.

Several patterns of chemical and structural transformations of lignin in spruce wood during thermal modification and accelerated aging are identified from this research that provide insight on how lignin responds to different thermal treatments and aging conditions, highlighting the trade-offs between depolymerization for product recovery and condensation for structural stability:

- Depolymerization at lower temperatures is explained by reactions involving the cleavage of β-*O*-4 bonds, resulting in a decreased molecular weight and an increased yield of degradation products, such as vanillin and vanillic acid.
- Condensation at higher temperatures (i.e., 210 °C) leads to an increased molecular weight and structural stability and, conversely, a decreased yield of NBO products due to the reduced cleavage of lignin inter-unit bonds.
- Accelerated aging results in a relative increase in the lignin content due to the leaching of water-soluble extractives; lignin in aged samples has a reduced molecular weight (Mw) compared with thermally treated samples, indicating further degradation and yielding higher amounts of aromatic monomers; aging also results in lower decomposition temperatures, which improves its suitability for further processing.
- Vanillin yield is highest at lower modification temperatures (160 °C) and decreases with increasing temperature; a strong negative correlation (–0.9255) shows that lower molecular weight lignin produces more vanillin.

## Figures and Tables

**Table 1:** The content of extractives, lignin, and dioxane lignin yields in untreated, thermally treated, and aged spruce wood (% odw, SD are in the parentheses).

*T* (°C)	Extractives (%)		Lignin (%)		Dioxane Lignin Yields			
	TW	TW-XE	TW	TW-XE	(% Wood)		(% Klason Lignin)	
					TW	TW-XE	TW	TW-XE
REF	0.98 (0.05)	0.98 (0.05)	26.24 (0.04)	26.24 (0.04)	4.75 (0.21)	4.75 (0.21)	18.11 (0.78)	18.11 (0.78)
160	2.51 (0.20)	1.63 (0.10)	26.45 (0.07)	27.68 (0.40)	5.38 (0.15)	5.15 (0.21)	20.32 (0.57)	18.59 (0.88)
180	2.71 (0.26)	2.25 (0.14)	28.65 (0.09)	32.14 (0.18)	7.68 (0.29)	8.40 (0.41)	26.81 (1.03)	26.16 (1.28)
210	3.49 (0.25)	3.07 (0.23)	33.08 (0.05)	35.26 (0.43)	9.13 (0.22)	9.92 (0.18)	27.58 (0.63)	28.13 (0.18)

**Table 2:** The content of nitrobenzene oxidation products yields in untreated, thermally treated, and aged spruce wood (% based on Klason lignin, SD are in the parentheses).

*T* (°)	TW			TW-XE		
	Vanillin	Vanillic Acid	SUM	Vanillin	Vanillic Acid	SUM
REF	21.75 (0.28)	1.53 (0.02)	23.28 (0.29)	21.75 (0.28)	1.53 (0.02)	23.28 (0.29)
160	23.75 (0.36)	1.68 (0.05)	25.43 (0.60)	25.09 (0.23)	2.00 (0.03)	27.09 (0.26)
180	21.29 (0.27)	2.18 (0.08)	23.47 (0.34)	24.23 (0.11)	1.62 (0.01)	25.85 (0.10)
210	18.47 (0.26)	0.81 (0.01)	19.28 (0.25)	18.97 (0.24)	1.33 (0.04)	20.31 (0.28)

**Table 4:** TG and DTG data on thermally treated wood.

Sample	Peak Temperature (°C)/Mass Loss (%)			Residual Mass (%)
	First Region	Second Region	Third Region	
REF	83/1.89	371/30.76	395–600/19.45	47.95
160-TW	60/2.11	369/31.94	395–600/19.57	46.39
180-TW	64/2.31	366/31.22	395–600/19.27	47.18
210-TW	64/2.31	374/24.36	395–600/19.27	52.90
160-TW-XE	62/2.08	344/27.39	392–600/22.36	48.11
180-TW-XE	64/2.22	346/24.47	393–600/24.42	48.86
210-TW-XE	66/2.50	360/20.26	392–600/27.25	49.97

**Table 5:** Correlation coefficients of individual dependencies of measured parameters.

Variable	VAN	VANac	*M*_n_	*M*_w_	*M*_z_	PDI
VAN	1.000000	0.664343	0.039300	−0.925533	−0.787225	−0.626182
VANac	0.664343	1.000000	−0.259399	−0.727719	−0.886412	−0.246322
*M*_n_	0.039300	−0.259399	1.000000	0.161560	0.459996	−0.769325
*M*_w_	−0.925533	−0.727719	0.161560	1.000000	0.869334	0.505147
*M*_z_	−0.787225	−0.886412	0.459996	0.869334	1.000000	0.154899
PDI	−0.626182	−0.246322	−0.769325	0.505147	0.154899	1.000000

## Data Availability

The original contributions presented in the study are included in the article, further inquiries can be directed to the corresponding author.
